# Concomitant Polytrauma in Fall-Related Proximal Humerus Fractures: A Fracture-Subtype-Stratified Analysis of In-Hospital Outcomes

**DOI:** 10.3390/jcm15166210

**Published:** 2026-08-11

**Authors:** Abdullah Raizah

**Affiliations:** Orthopedic Department, College of Medicine, King Khalid University, Abha 62521, Saudi Arabia; araizah@kku.edu.sa

**Keywords:** proximal humerus fracture, polytrauma, Injury Severity Score, in-hospital mortality, non-home discharge, propensity score matching, geriatric trauma

## Abstract

**Objectives**: To determine whether concomitant polytrauma is independently associated with worse in-hospital outcomes among fall-related proximal humerus fracture (PHF) patients and whether fracture subtype is associated with heterogeneity in this association. **Methods**: Retrospective cohort study using the American College of Surgeons Trauma Quality Program Participant Use File (ACS TQP-PUF; 2019–2022). Adults with PHF and a fall mechanism were included. Multivariable logistic regression adjusted for age, sex, the five mFI-5 components, anticoagulant use, and calendar year. Non-home discharge (NHD) was restricted to survivors. Sensitivity analyses used 1:1 PSM (all primary covariates in propensity model; post-matching SMDs assessed) and restriction to ground-level falls. **Results**: Among 63,995 fall-related PHF patients (70.5% female; 75.8% aged ≥65 years), 3884 (6.1%) met polytrauma criteria (ISS ≥ 16); polytrauma patients were more often male (45.1% vs. 28.5%) and slightly younger (mean age 69.3 vs. 72.0 years). Polytrauma was independently associated with in-hospital mortality (aOR 7.05, 95% CI 6.14–8.09) and non-home discharge among survivors (aOR 1.95, 95% CI 1.79–2.12; crude 72.0% vs. 64.6%). ICU utilization (aOR 13.25, 95% CI 12.34–14.22) and any complication (aOR 3.41, 95% CI 3.07–3.79) are reported as descriptive markers subject to triage and ascertainment bias, respectively. The ICD-10 subtype-stratified mortality aORs ranged from 3.36 to 8.28 across five strata (exploratory; no formal interaction test performed). PSM (3884 pairs; *n* = 7768) and ground-level fall restriction produced estimates consistent with the primary analyses. **Conclusions**: Concomitant polytrauma was independently associated with substantially higher in-hospital mortality and non-home discharge after fall-related PHF, identifying a high-risk subgroup that warrants closer clinical attention.

## 1. Introduction

Proximal humerus fractures rank among the three most frequent fractures in adults over 65 years of age, accounting for approximately 5–6% of all emergency department fracture presentations [1,2,3]. The annual incidence continues to rise in aging populations worldwide [2,4]. Humeral fractures have negative outcomes comparable to hip fractures in this population [5,6].

Outcomes following PHF are shaped by patient-level factors beyond the fracture. Clement et al. reported a 10% one-year mortality rate in 637 elderly patients [7]. Van Grootven et al. validated a six-variable prediction model in 1265 Belgian patients [8]. Cheng et al. queried the Nationwide Inpatient Sample in 137,810 geriatric PHF patients [9]. None of the studies examined concomitant polytrauma as an independent outcome determinant. Operative management of PHF with plate fixation versus intramedullary nailing carries similar perioperative outcomes [10], but geriatric trauma guidelines advocate early orthogeriatric co-management for all injury patterns [11], an approach whose application in polytrauma PHF patients has not been studied.

Polytrauma affects fracture outcomes through hemodynamic instability, competing surgical priorities, and prolonged immobilization [12,13]. Existing analyses combine all injury mechanisms, conflating high-energy motor vehicle collisions with low-energy ground-level falls. However, whether the polytrauma burden varies across the PHF subtype groups remains unclear. We conducted a mechanism-restricted retrospective cohort study to quantify the frailty-adjusted association between polytrauma and in-hospital outcomes in fall-related PHF, explore heterogeneity across fracture subtype groups, and assess robustness through PSM, age-stratified analysis, and ground-level fall restriction.

## 2. Methods

### 2.1. Study Design and Data Source

This retrospective cohort study was reported in accordance with the STROBE guideline [14]. Because the data were de-identified and contained no identifiable private information, the study did not constitute human-subjects research under 45 CFR 46.102(e); institutional review board review and approval were therefore not required. Data were obtained from the American College of Surgeons (ACS) Trauma Quality Program Participant Use File (TQP-PUF; 2019–2022) [15].

### 2.2. Study Population

Adults ≥18 years with ICD-10 PHF codes (S42.20–S42.29) and fall mechanism (W00–W19, encompassing ground-level [W00–W09] and height-related falls [W10–W19]). Non-fall mechanisms were excluded. Polytrauma: ISS ≥ 16; isolated PHF: ISS < 16. Age stratification was performed at 65 years [16].

### 2.3. Covariates and Frailty

The mFI-5 (functional dependence, COPD, CHF, hypertension, and diabetes) was the primary comorbidity measure [17]. Regression models were adjusted for mFI-5 (five binary components), age (continuous, linear), sex, anticoagulant use, and the calendar year. ISS was excluded because it defined the exposure groups. The ACS verification level is reported descriptively in Table 1 only and was not included as a regression covariate; it was missing in 27.6% of the cohort, but because it was never a model covariate, this missingness does not affect the analytic sample size (*N* = 63,995 in all primary models).

### 2.4. Outcomes

The primary outcome was in-hospital mortality. Any in-hospital complication and ICU admission were captured as exploratory, descriptive outcomes; they are subject to differential ascertainment bias and triage-driven admission, respectively, and were not treated as co-equal outcomes. Any in-hospital complication was a composite that included the following registry-recorded events: unplanned ICU admission, unplanned intubation, delirium, unplanned return to the operating room, cardiac arrest, acute kidney injury, deep vein thrombosis, stroke or cerebrovascular accident, acute respiratory distress syndrome, pulmonary embolism, sepsis, myocardial infarction, and superficial and deep incisional surgical site infection.

Non-home discharge (NHD) was the secondary outcome and was restricted to survivors. We restricted the NHD regression and crude rate calculations for patients with mortality = 0 (*n* = 62,897). No patients among the survivors had missing discharge disposition data; therefore, no additional exclusions were required.

### 2.5. ICD-10 Fracture Subtype Analysis

PHF subtypes were grouped into five ICD-10 strata based on clinical and coding rationale: (1) greater tuberosity fractures (S42.25): single-fragment, typically conservatively managed; (2) 2-part surgical neck fractures (S42.22): most common PHF pattern, defined fracture line at the surgical neck; (3) surgical neck, not otherwise specified (S42.21): unspecified coding, which may reflect centers with less granular coding practices or fracture patterns that do not conform to a standard 2-part description; (4) 3-/4-part complex fractures (S42.23 and S42.24): multi-fragment patterns with higher likelihood of operative management and perioperative complexity; and (5) “Other/Unspecified”, which included unspecified fractures of the upper end of the humerus (S42.20), torus fractures (S42.27), other fractures of the upper end of the humerus (S42.29), and the small number of lesser tuberosity fractures (S42.26; *n* = 209) too infrequent to analyze separately. Patients carrying more than one PHF code were assigned their most severe subtype, using the hierarchy 4-part > 3-part > 2-part > surgical neck NOS > greater tuberosity > lesser tuberosity > other/unspecified. This stratum accounted for 26,715 patients (41.7% of the cohort; 1746 of 3884 polytrauma patients) and was included in all subtype-stratified analyses below to avoid undisclosed exclusion of a substantial portion of the cohort.

### 2.6. Sensitivity Analyses

The 1:1 nearest-neighbor PSM [18] (caliper 0.2 SD logit propensity score [19], no replacement) followed Gogna et al. [20]. The propensity model included all primary regression covariates: age (continuous), sex, five mFI-5 components (functional dependence, COPD, CHF, hypertension, and diabetes), anticoagulant use, and calendar year. The ACS verification level was not included in the propensity model, consistent with its exclusion from the outcome regressions (see Section 2.3). Post-matching covariate balance was assessed using standardized mean differences (SMDs) [21]; all modeled covariates achieved SMD < 0.10 after matching. Weighted logistic regression with robust standard errors was applied to the matched sample for each modeled outcome. Additional sensitivity analyses were performed by (1) restricting the analysis to ground-level falls only (W00–W09) to confirm that the associations were not driven by higher-energy fall mechanisms within the W00–W19 category and (2) using age-stratified models (<65 vs. ≥65 years) and stratum-specific aORs without formal interaction testing.

### 2.7. Statistical Analysis

Age was modeled as a continuous linear term in all the regression models. Model discrimination was assessed using the area under the receiver operating characteristic curve (C-statistic), and calibration of the primary mortality model was assessed using the Hosmer–Lemeshow goodness-of-fit test (10 groups). Continuous variables were summarized as medians with IQRs and compared using Wilcoxon tests; categorical variables were compared using chi-square tests. NHD rates and regression were restricted to survivors (*n* = 62,897), as described above. Length of stay (LOS) was complete for all 63,995 patients (0% missing) and was used descriptively only; it was not included as a regression covariate. For display only, the length-of-stay box plot was truncated at 29 days (99th percentile); 0.9% of observations exceeded the displayed range. Box and whiskers were calculated from the full available data. All analyses were performed using R version 4.3.3. All *p*-values were two-sided, with α = 0.05.

## 3. Results

### 3.1. Study Population

From the ACS TQP-PUF (2019–2022), 63,995 fall-related PHF patients were identified: 3884 (6.1%) with polytrauma and 60,111 (93.9%) with isolated PHF. The cohort was 70.5% female and 75.8% aged ≥65 years. Polytrauma patients were more often male (45.1% vs. 28.5%), slightly younger (mean age 69.3 ± 15.9 vs. 72.0 ± 12.7 years), had higher ISS (median 21 vs. 5), and were treated at Level I centers more frequently (64.1% vs. 47.8%, among patients with available ACS verification level data; Table 1). The median LOS was longer in the polytrauma group (7 vs. 5 days), and the LOS was complete for all patients (0% missing).

### 3.2. Associated-Injury Profile (Polytrauma Group)

Among polytrauma patients, AIS ≥ 3 injury was present, as a proportion of the full polytrauma cohort (missing values treated as not meeting the threshold, yielding a conservative estimate), in 60.2% by head/neck region, 40.2% by chest, 24.4% by extremities, 9.8% by abdomen, 1.4% by face, and 0.5% by external regions. Region-specific data completeness varied (missingness ranged from <1% for extremities to 71.9% for abdomen); among patients with non-missing data for each region, rates were correspondingly higher (e.g., 84.4% for head/neck, 74.2% for chest), so the figures above should be read as lower-bound estimates. The median number of non-extremity body regions with AIS ≥ 2 injury was 2 (IQR 1–2) in the polytrauma group versus 0 (IQR 0–0) in the isolated PHF group. Polytrauma patients were more often injured by height-related falls (ICD-10 W10–W19: 58.2%) than ground-level falls (W00–W09: 38.0%), the reverse of the isolated PHF group (38.2% height-related vs. 60.7% ground-level), consistent with polytrauma reflecting greater injury energy rather than a shoulder-specific phenomenon.

### 3.3. Mortality and Exploratory In-Hospital Outcomes

**Figure 1:**
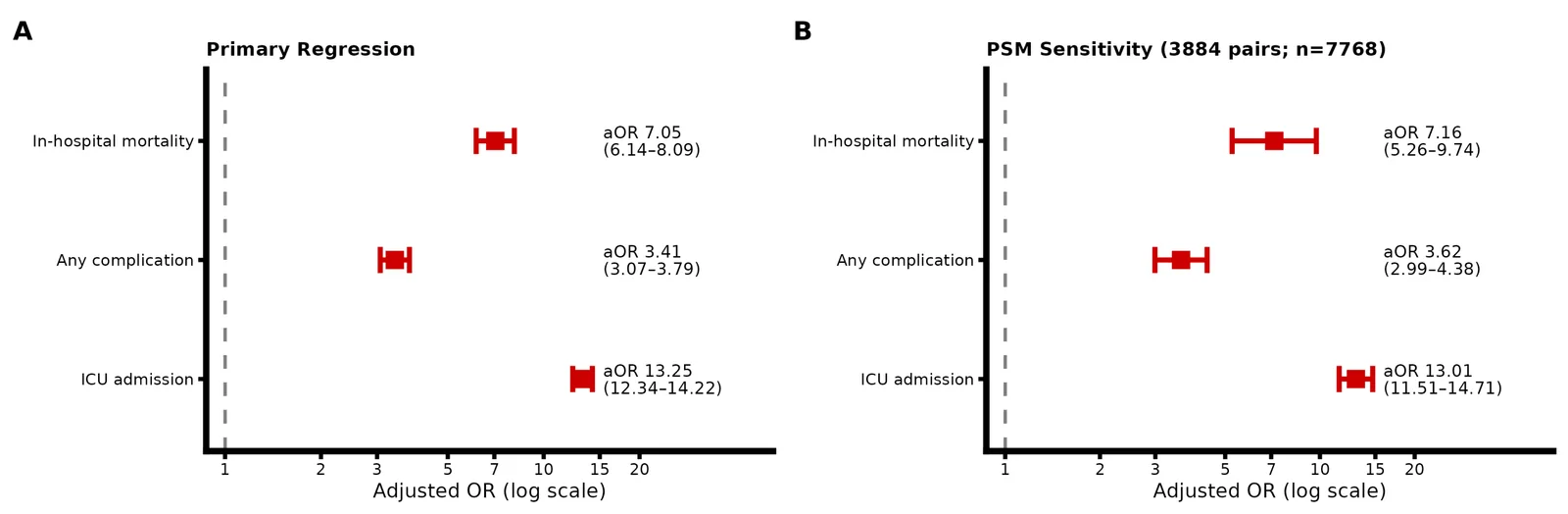
Primary regression and PSM sensitivity. Panel (**A**): primary regression (*n* = 63,995). Panel (**B**): PSM (3884 matched pairs; *n* = 7768; post-matching SMDs < 0.10 for all covariates; weighted logistic regression). Log scale.

**Figure 2:**
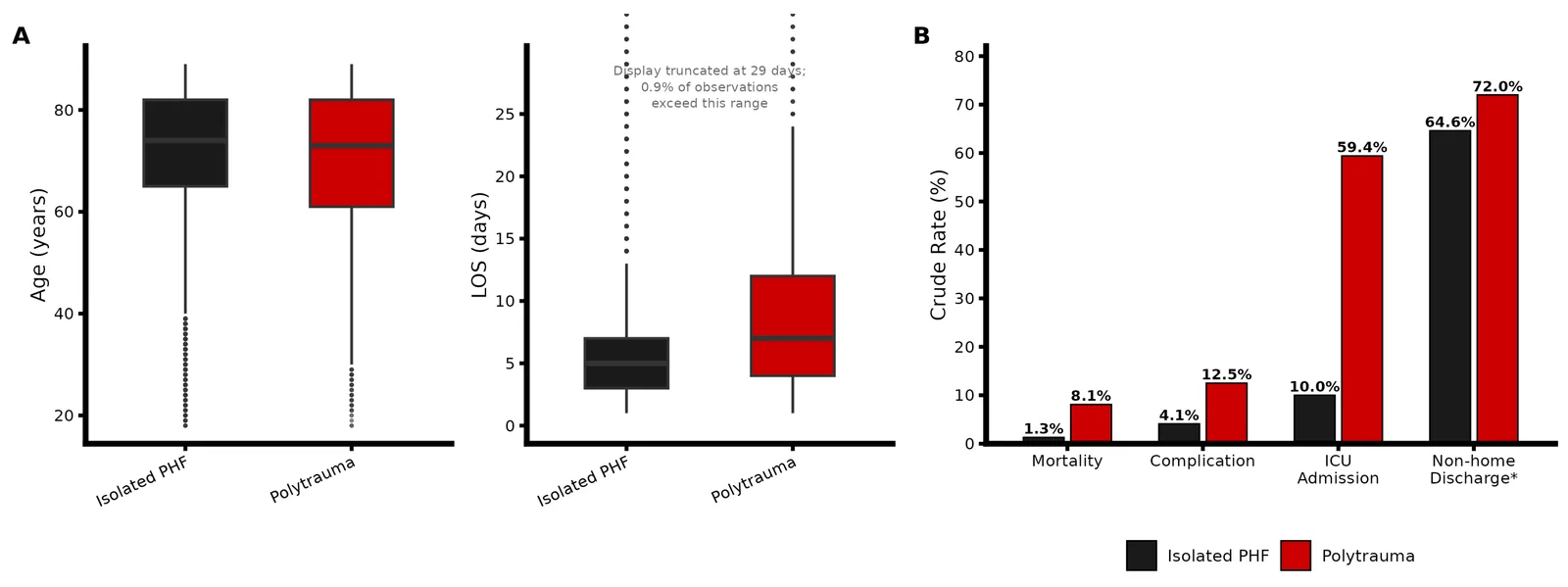
Baseline distributions and outcome rates. Panel (**A**): box plots of age and LOS. Panel (**B**): outcome rates. NHD (*) was restricted to survivors (72.0% polytrauma vs. 64.6% isolated PHF).

After multivariable adjustment (Table 2, Figure 1A), polytrauma was associated with increased mortality (aOR 7.05, 95% CI 6.14–8.09; *p* < 0.001), complications (aOR 3.41, 95% CI 3.07–3.79; *p* < 0.001), and ICU resource utilization (aOR 13.25, 95% CI 12.34–14.22; *p* < 0.001). Crude mortality was 8.1% vs. 1.3%, crude complication 12.5% vs. 4.1%, and crude ICU 59.4% vs. 10.0% (Figure 2). The primary mortality model showed adequate discrimination (C-statistic 0.760) but statistically detectable miscalibration on the Hosmer–Lemeshow test (χ^2^ = 25.88, df = 8, *p* = 0.001), a common finding in large administrative registry samples, where this test is highly sensitive to minor deviations from perfect calibration, even when discrimination and effect estimates are stable.

**Table 2:** Multivariable-adjusted outcomes: primary and sensitivity analyses.

Outcome	Analysis	aOR (95% CI)	*p*	*N*
In-hospital mortality	Primary regression	7.05 (6.14–8.09)	<0.001	63,995
	PSM sensitivity ^†^	7.16 (5.26–9.74)	<0.001	7768
	Ground-level falls	5.40 (4.31–6.76)	<0.001	37,955
Any complication ^‡^	Primary regression	3.41 (3.07–3.79)	<0.001	63,995
	PSM sensitivity ^†^	3.62 (2.99–4.38)	<0.001	7768
	Ground-level falls	2.60 (2.18–3.11)	<0.001	37,955
ICU admission (acuity/resource utilization marker) ^‡^	Primary regression	13.25 (12.34–14.22)	<0.001	63,995
	PSM sensitivity ^†^	13.01 (11.51–14.71)	<0.001	7768
Non-home discharge ^§^	Main analysis (survivors)	1.95 (1.79–2.12)	<0.001	62,897

^†^ PSM: 3884 matched pairs; *n* = 7768. Propensity model covariates included age, sex, functional dependence, COPD, CHF, hypertension, diabetes, anticoagulant use, and calendar year. Post-matching SMDs < 0.10 for all modeled covariates (full balance table in Table 3). Weighted logistic regression with robust standard errors. ^‡^ Complication estimates are subject to differential ascertainment bias because of longer LOS in the polytrauma group. ICU-admission estimates should be interpreted as markers of acuity and triage-driven resource utilization. ^§^ Secondary outcome; restricted to survivors (*n* = 62,897); in-hospital decedents coded discharge = 0 were excluded; no missing discharge data among survivors; crude NHD 72.0% vs. 64.6% (*p* = 1.3 × 10^−19^). Primary and ground-level-fall regression models were adjusted for the five mFI-5 components, age, sex, anticoagulant use, and calendar year. The same covariates were included in the PSM propensity model. The ACS verification level was not a model covariate in any analysis (reported descriptively only; see Section 2). Primary mortality model: C-statistic 0.760; Hosmer–Lemeshow χ^2^ = 25.88 (df = 8), *p* = 0.001 (see Section 4 for the interpretation).

**Table 3:** Sensitivity analyses and PSM post-matching covariate balance.

**Part A. Ground-Level Falls Sensitivity Analysis (*n* = 37,955; polytrauma *n* = 1477)**				
Outcome	aOR (95% CI)	*p*		
In-hospital mortality	5.40 (4.31–6.76)	<0.001		
Any complication	2.60 (2.18–3.11)	<0.001		
ICU admission	12.91 (11.56–14.43)	<0.001		
**Part B. PSM Post-Matching Covariate Balance**				
Covariate	Mean Polytrauma	Mean Isolated PHF	|SMD| after matching	
Propensity score (distance)	0.0705	0.0705	<0.001	
Age, years	69.3	69.5	0.012	
Sex (female)	0.549	0.548	0.002	
Functional dependence	0.192	0.182	0.024	
COPD	0.116	0.112	0.013	
Congestive heart failure	0.083	0.073	0.035	
Hypertension	0.567	0.574	0.015	
Diabetes	0.233	0.232	0.002	
Anticoagulant use	0.220	0.215	0.012	
Calendar year 2019	0.230	0.236	0.013	
Calendar year 2020	0.246	0.247	0.001	
Calendar year 2021	0.255	0.249	0.014	
Calendar year 2022	0.268	0.268	0.001	

Restricted to ICD-10 W00–W09 (ground-level falls only). The same covariate set was used for the primary analysis. All *p* < 0.001. Note: 1:1 nearest-neighbor matching, caliper 0.2 SD logit propensity score; *n* = 7768 total; 3884 matched pairs.

### 3.4. Non-Home Discharge

After explicitly excluding in-hospital deaths (*n* = 1098, all coded discharge = 0 in the source data), the number of survivors was 62,897. The crude NHD was 72.0% (polytrauma) vs. 64.6% (isolated PHF; *p* = 1.3 × 10^−19^). After multivariable adjustment, the aOR was 1.95 (95% CI 1.79–2.12; *p* < 0.001). Age-stratified NHD aORs were 2.62 (95% CI: 2.30–2.99) for <65 years and 1.58 (95% CI: 1.42–1.75) for ≥65 years (both *p* < 0.001), indicating a significant polytrauma association with non-home discharge in both age strata.

### 3.5. ICD-10 Subtype-Stratified Analysis (Exploratory)

**Figure 3:**
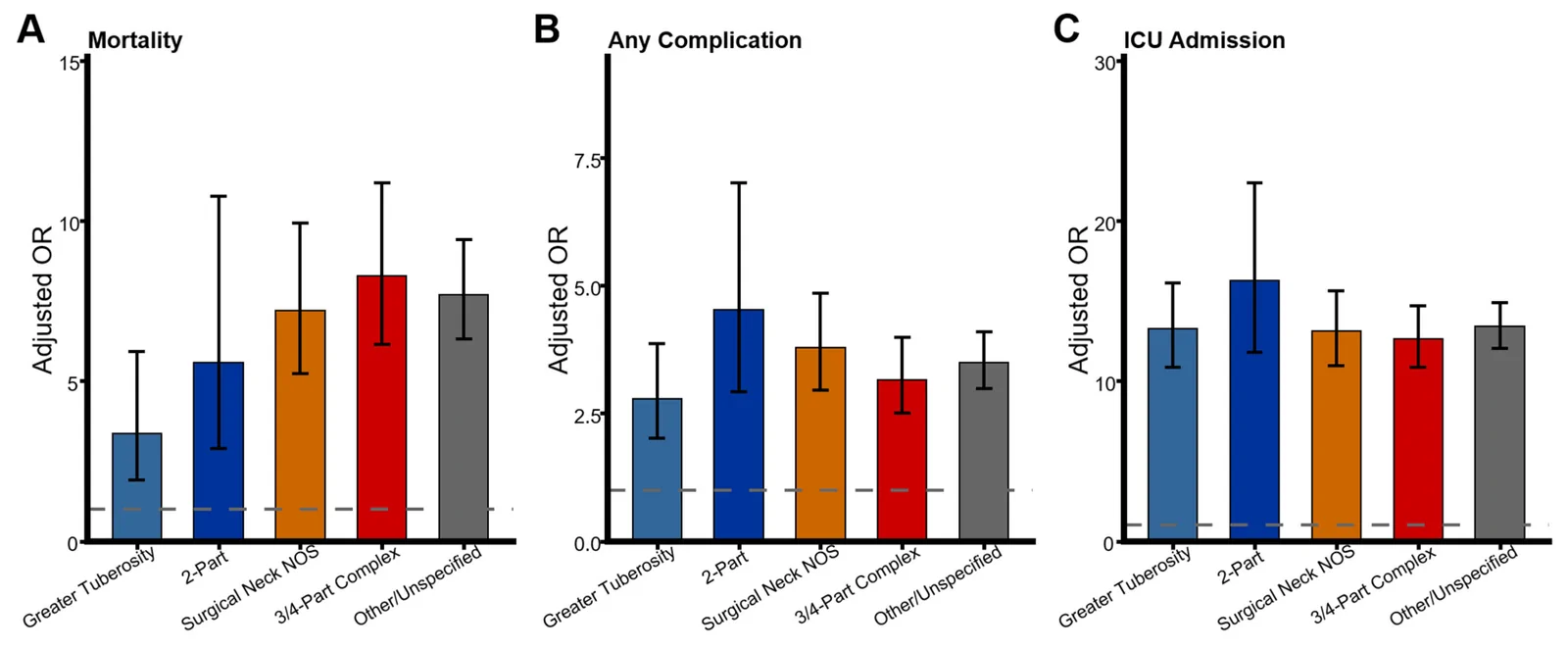
ICD-10 subtype-stratified adjusted odds ratios (exploratory). Separate models within each of the five ICD-10 strata, including a fifth “Other/Unspecified” stratum (41.7% of the cohort; see Section 2); no formal interaction test was performed. Panel (**A**): mortality. Panel (**B**): complications. Panel (**C**): ICU acuity. Error bars = 95% CI. The dashed line indicates the null value (aOR = 1.0).

Separate stratum-specific models were fitted across five predefined ICD-10 strata (Figure 3A). This analysis was exploratory and hypothesis-generating, and no formal interaction test was performed. The adjusted mortality odds ratios were as follows: greater tuberosity aOR 3.36 (95% CI 1.91–5.91; 521 polytrauma [16 deaths] vs. 7180 isolated [69 deaths]), 2-part aOR 5.56 (95% CI 2.88–10.75; 195 [13] vs. 3470 [48]), surgical neck NOS aOR 7.19 (95% CI 5.22–9.92; 596 [58] vs. 10,500 [167]), 3-/4-part complex aOR 8.28 (95% CI 6.13–11.18; 826 [69] vs. 13,992 [155]), and Other/Unspecified aOR 7.69 (95% CI 6.30–9.40; 1746 [160] vs. 24,969 [343]). Confidence intervals overlapped for most pairs of strata; the greater tuberosity interval did not overlap those of the 3-/4-part complex or Other/Unspecified strata. No formal interaction test was performed, and between-stratum differences should not be interpreted as established effect modification. The surgical neck NOS and Other/Unspecified estimates both require cautious interpretation given coding heterogeneity; the latter stratum accounts for 41.7% of the cohort and is analyzed rather than excluded to avoid an undisclosed exclusion of a substantial portion of the sample. Complications and ICU aORs showed a similar pattern across the strata (Figure 3B,C).

### 3.6. Age-Stratified Analysis

**Figure 4:**
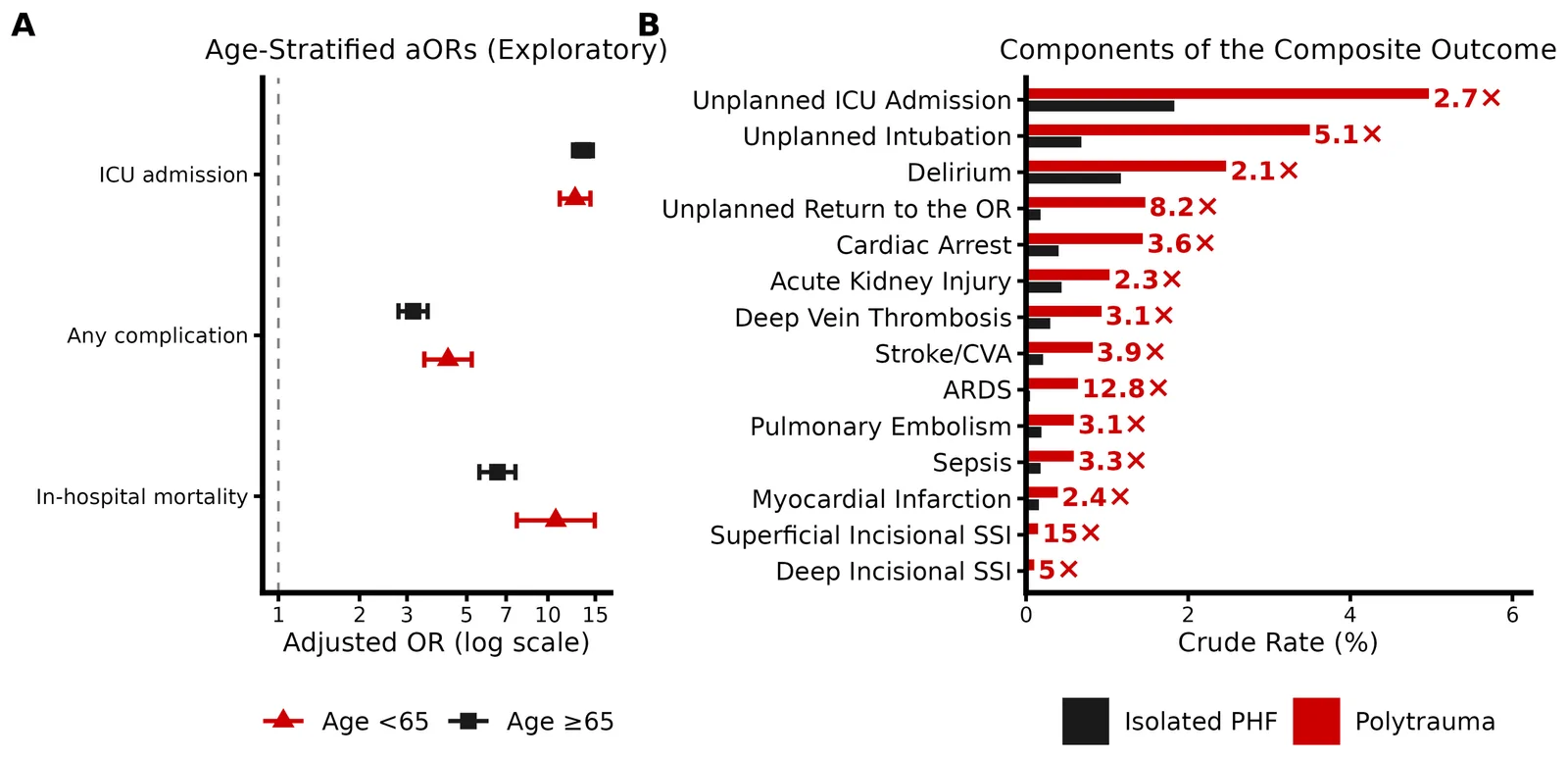
Age-stratified outcomes (exploratory) and individual complication rates. Panel (**A**): age-stratified aORs (separate models; no formal interaction test). Panel (**B**): crude rates of the individual components of the composite complication outcome, subject to differential ascertainment bias (see Section 4). Values beside the bars are crude rate ratios (polytrauma relative to isolated PHF), e.g., 2.7×.

The mortality aOR was 10.70 (95% CI 7.67–14.93) for <65 years and 6.50 (95% CI 5.57–7.58) for ≥65 years (Figure 4A).

### 3.7. Sensitivity Analyses

PSM (3884 matched pairs, *n* = 7768). Post-matching SMDs were below 0.10 for all the modeled covariates (Table 3). Matched estimates: mortality aOR 7.16 (95% CI 5.26–9.74), complications aOR 3.62 (95% CI 2.99–4.38), and ICU acuity aOR 13.01 (95% CI 11.51–14.71; Figure 1B). Ground-level fall restriction (*n* = 37,955; polytrauma *n* = 1477): mortality aOR 5.40 (95% CI 4.31–6.76), complications aOR 2.60 (95% CI 2.18–3.11), and ICU admission aOR 12.91 (95% CI 11.56–14.43).

## 4. Discussion

In this frailty-adjusted analysis of 63,995 fall-related PHF patients, concomitant polytrauma was associated with approximately sevenfold higher odds of in-hospital mortality, 3.4-fold higher odds of any complication, 13-fold higher odds of ICU admission, and nearly twofold higher odds of non-home discharge among survivors. Subtype-specific mortality associations varied across the five strata, although no formal interaction test was performed. The PSM and ground-level fall restriction produced estimates consistent with the primary analysis.

The strong association observed for polytrauma complements previous studies identifying age, sex, frailty, and individual comorbidities as prognostic factors after PHF. Direct numerical comparison with previously reported hazard ratios or model-discrimination statistics is limited because these measures address different estimands. Clement et al. identified hazard ratios of 1.1–2.8 for individual comorbidity predictors [7]. Van Grootven et al. achieved an AUC of 0.81 without polytrauma among the predictor candidates [8]. Other studies have identified age, male sex, and comorbidity burden as mortality predictors in older patients with PHFs [22], and post-trauma mortality has been observed to increase sharply from age 60 [23], consistent with the larger polytrauma effect seen in younger patients in our cohort. Concordance between primary regression (aOR 7.05) and PSM (aOR 7.16) supports robustness to measured confounding; residual confounding from unmeasured variables cannot be excluded from this study.

To our knowledge, no prior study has examined stratum-specific polytrauma associations across PHF subtype groups. One possible explanation is that greater tuberosity fractures, which are conservatively managed and mechanically simpler, may permit earlier mobilization even when polytrauma is present [24], whereas 3-/4-part patterns requiring operative fixation create competing priorities with other injury-related interventions [25]. However, this mechanistic account remains unconfirmed because fracture complexity in a fall-related cohort is itself a proxy for injury energy, and the between-stratum differences may instead reflect confounding by injury mechanism rather than a shoulder-specific effect. This interpretation is consistent with our associated injury profile, in which polytrauma patients had a substantially higher AIS ≥ 3 burden in the head/neck and chest and were disproportionately injured by height-related rather than ground-level falls—injuries and mechanisms not captured by fracture subtype and not adjusted for in the stratified models. The surgical neck NOS and Other/Unspecified strata both require cautious interpretation given coding heterogeneity; the latter accounted for 41.7% of the cohort and was analyzed rather than excluded.

The rate of non-home discharge was significantly higher among polytrauma survivors across both age groups. Prior reports of a null NHD effect in elderly patients with falls may have been influenced by the inclusion of in-hospital deaths coded as NHD = 0 in the discharge-disposition denominator, which would attenuate the observed polytrauma effect. With correct survivor-only analysis, both age groups showed significant effects (aOR 2.62 for <65, 1.58 for ≥65; both *p* < 0.001), with a larger relative effect in younger patients reflecting their lower absolute baseline rate of non-home discharge [26].

The ICU aOR (13.25) should be interpreted as an acuity and resource utilization marker rather than a discrete adverse outcome. Polytrauma patients are admitted to the ICU in part per institutional triage protocols driven by injury severity, and the large aOR is partly expected because ISS ≥ 16 is generally associated with greater acuity and a higher likelihood of ICU-level care. Mortality in older trauma patients at verified centers has improved over time [27]; however, polytrauma remains a strong independent predictor in our cohort. Notably, blood transfusion and surgical treatment independently predict mortality in PHF [28], venous thromboembolism in upper extremity fractures is clinically significant [29], and non-surgical management of PHF carries a high burden of poor prognosis [30]. Ground-level fall restriction confirmed that associations were not explained by higher-energy fall mechanisms within the category (mortality aOR 5.40, ground-level vs. 7.05, all falls), consistent with an association not fully explained by differences in fall mechanism.

### Strengths and Limitations

The strengths of this study include the large multicenter registry, mechanism restriction, frailty adjustment, explicit correction of NHD dead-patient coding with full transparency, PSM with post-matching SMD assessment, and ground-level fall restriction.

Limitations include the following: (1) ACS TQP-PUF captures in-hospital events only [15,31]; (2) ISS ≥16 without physiological component; (3) ICD-10 does not map onto Neer classification; surgical neck NOS and Other/Unspecified codes may reflect coding practice variation; (4) W00–W19 includes height-related falls (stairs and ladder falls are included in ICD-10 W10–W19) [32,33], and ground-level restriction confirmed robustness; (5) trauma-center-only patients may overrepresent severe cases; (6) length of stay was complete in this cohort (0% missing) and was used descriptively only, not as a regression covariate; (7) differential ascertainment bias for complications: polytrauma patients have longer LOS and higher ICU rates, creating greater opportunity to detect complications regardless of true event frequency; this bias inflates the complication aOR estimate and readers should apply conservative interpretation to this outcome specifically; the composite comprised the complication fields available in the analytic dataset and did not include ventilator-associated pneumonia, pressure ulcer, extremity compartment syndrome, catheter-associated urinary tract infection, central line-associated bloodstream infection, osteomyelitis, or organ/space surgical site infection, so it should be read as a partial rather than an exhaustive complication inventory; (8) residual confounding from injury severity components not captured by ISS (e.g., AIS region profiles, physiological derangement), pre-injury functional status, and medications beyond anticoagulants cannot be excluded; (9) subtype-stratified and age-stratified models are exploratory; formal interaction testing is required to establish effect modification; (10) exclusion of non-fall mechanisms limits generalizability to the high-energy trauma population; (11) ACS verification level was missing in 27.6% of the cohort and is reported descriptively in Table 1 only, and because it was not a regression covariate, this missingness does not affect any model’s analytic sample; and (12) the associated-injury (AIS) profile has region-specific missingness ranging from <1% to 71.9% and should be interpreted as a lower-bound, indicative estimate rather than a complete injury inventory.

## 5. Conclusions

Among patients with fall-related PHF, concomitant polytrauma was independently associated with substantially higher in-hospital mortality and non-home discharge after frailty adjustment. Associations with complications and ICU resource utilization are reported descriptively, given the differential ascertainment bias and the triage-driven nature of ICU admission, respectively, and should not be interpreted as co-equal outcomes. The PSM and ground-level fall restriction produced estimates consistent with the primary analysis.

## Figures and Tables

**Table 1:** Baseline characteristics of fall-related PHF patients (*N* = 63,995).

Characteristic	Isolated PHF (*n* = 60,111)	Polytrauma (*n* = 3884)	SMD
Age, median (IQR); mean (SD)	74.0 (65.0–82.0); 72.04 (12.71)	73.0 (61.0–82.0); 69.32 (15.87)	0.19
Female sex, %	42,956 (71.5)	2133 (54.9)	0.35
Diabetes, %	16,982 (28.3)	906 (23.3)	0.11
Hypertension, %	38,410 (63.9)	2201 (56.7)	0.15
COPD, %	8558 (14.2)	451 (11.6)	0.08
CHF, %	5635 (9.4)	322 (8.3)	0.04
Functional dependence, %	13,826 (23.0)	744 (19.2)	0.09
Anticoagulant use, %	13,076 (21.8)	853 (22.0)	0.01
ISS, median (IQR) ^†^	5.0 (4.0–9.0)	21.0 (17.0–24.0)	3.18
LOS, days, median (IQR) ^‡^	5.0 (3.0–7.0)	7.0 (4.0–12.0)	0.48
Level I trauma center, % ^§^	20,746/43,429 (47.8)	1869/2918 (64.1)	0.33
Mortality, %	782 (1.3)	316 (8.1)	0.33
Any complication, %	2491 (4.1)	485 (12.5)	0.31
ICU admission (resource utilization), %	6040 (10.0)	2309 (59.4)	1.21
Non-home discharge, % (survivors) ^¶^	38,304/59,329 (64.6)	2570/3568 (72.0)	--

^†^ ISS defines exposure; excluded from regression analysis. ^‡^ LOS complete for all patients (0% missing); the box plot display was truncated at 29 days (99th percentile); 0.9% of observations exceeded the displayed range. ^§^ ACS verification level available for 43,429 of 60,111 isolated PHF patients (72.2%) and 2918 of 3884 polytrauma patients (75.1%); percentages calculated within patients with non-missing verification level data; verification level is reported descriptively only and was not included as a regression covariate (see Section 2.3). ^¶^ NHD denominator restricted to survivors (mortality = 0; isolated *n* = 59,329; polytrauma *n* = 3568). In-hospital decedents (*n* = 1098; 782 isolated, 316 polytrauma) were coded discharge = 0 in the TQP-PUF source data and excluded prior to analysis. No survivors had missing discharge information. Chi-square *p* < 0.001. SMD ≥ 0.10 = meaningful imbalance.

## Data Availability

The underlying data are available to eligible investigators through the ACS Trauma Quality Program (https://www.facs.org/quality-programs/trauma/tqp, accessed on 1 August 2026).

## Abbreviations

Abbreviation	Definition
ACS	American College of Surgeons
aOR	Adjusted Odds Ratio
AIS	Abbreviated Injury Scale
AKI	Acute Kidney Injury
AUC	Area Under the (Receiver Operating Characteristic) Curve
CHF	Congestive Heart Failure
CI	Confidence Interval
COPD	Chronic Obstructive Pulmonary Disease
DVT	Deep Vein Thrombosis
ICD-10	International Classification of Diseases, 10th Revision
ICU	Intensive Care Unit
IQR	Interquartile Range
IRB	Institutional Review Board
ISS	Injury Severity Score
LOS	Length of Stay
mFI-5	Modified Frailty Index-5
NHD	Non-Home Discharge
NOS	Not Otherwise Specified
OR	Odds Ratio
PE	Pulmonary Embolism
PHF	Proximal Humerus Fracture
PSM	Propensity Score Matching
SD	Standard Deviation
SMD	Standardized Mean Difference
STROBE	Strengthening the Reporting of Observational Studies in Epidemiology
TQP-PUF	Trauma Quality Program Participant Use File
VAP	Ventilator-Associated Pneumonia
